# Enhancing laboratory capacity during Ebola virus disease (EVD) heightened surveillance in Liberia: lessons learned and recommendations

**DOI:** 10.11604/pamj.supp.2019.33.2.17366

**Published:** 2019-05-29

**Authors:** Victoria Katawera, Henry Kohar, Nuha Mahmoud, Philomena Raftery, Christine Wasunna, Ben Humrighouse, Patrick Hardy, John Saindon, Randal Schoepp, Monear Makvandi, Lisa Hensley, Orla Condell, Kara Durski, Shalini Singaravelu, Laetitia Gahimbare, Gene Olinger, Francis Kateh, Dhamari Naidoo, Peter Nsubuga, Pierre Formenty, Tolbert Nyenswah, Sheick Oumar Coulibaly, Joseph Chukwudi Okeibunor, Ambrose Talisuna, Ali Ahmed Yahaya, Soatiana Rajatonirina, Desmond Williams, Bernice Dahn, Alex Gasasira1, Ibrahima Socé Fall

**Affiliations:** 1 World Health Organization, Monrovia, Liberia; 2 Ministry of Health, Monrovia, Liberia; 3 Formarly Academic Consortium Combating Ebola in Liberia, Monrovia, Liberia; 4 United States Centers for Disease Control and Prevention, Atlanta, United States of America; 5 United States Army Medical Research Institute of Infectious Diseases, Fort Detrick, Maryland, United States of America; 6 Sandia National Laboratories, Albuquerque, New Mexico, United States of America; 7 National Institutes of Health,Bethesda, United States of America; 8 World Health Organization, Geneva, Switzerland; 9 Global Public Health Solutions, Atlanta, Georgia, United States of America; 10 World Health Organization, Regional Office for Africa, Brazzaville, Congo

**Keywords:** Laboratory capacity, Ebola Virus Disease, enhanced surveillance

## Abstract

**Introduction:**

Following a declaration by the World Health Organization that Liberia had successfully interrupted Ebola virus transmission on May 9th, 2015; the country entered a period of enhanced surveillance. The number of cases had significantly reduced prior to the declaration, leading to closure of eight out of eleven Ebola testing laboratories. Enhanced surveillance led to an abrupt increase in demand for laboratory services. We report interventions, achievements, lessons learned and recommendations drawn from enhancing laboratory capacity.

**Methods:**

Using archived data, we reported before and after interventions that aimed at increasing laboratory capacity. Laboratory capacity was defined by number of laboratories with Ebola Virus Disease (EVD) testing capacity, number of competent staff, number of specimens tested, specimen backlog, daily and surge testing capacity, and turnaround time. Using Stata 14 (Stata Corporation, College Station, TX, USA), medians and trends were reported for all continuous variables.

**Results:**

Between May and December 2015, interventions including recruitment and training of eight staff, establishment of one EVD laboratory facility, implementation of ten Ebola GeneXpert diagnostic platforms, and establishment of working shifts yielded an 8-fold increase in number of specimens tested, a reduction in specimens backlog to zero, and restoration of turn-around time to 24 hours. This enabled a more efficient surveillance system that facilitated timely detection and containment of two EVD clusters observed thereafter.

**Conclusion:**

Effective enhancement of laboratory services during high demand periods requires a combination of context-specific interventions. Building and ensuring sustainability of local capacity is an integral part of effective surveillance and disease outbreak response efforts.

## Introduction

One of the pillars of effective response to an outbreak is an efficient laboratory system [1]. Between March, 2014 [2, 3] and March, 2015 [4], Liberia battled what has been reported to be the largest Ebola virus epidemic in history [5, 6]. During this period, the country had up to 11 laboratories testing for Ebola Virus Disease (EVD) with support from the government of Liberia (GoL), the World Health Organization (WHO) and international partners [1]. On May 9 2015, the WHO made a declaration that Liberia had successfully interrupted transmission of Ebola virus, following a 42 day period since the death of the last EVD patient in that outbreak [4]. There, however, continued to be a risk of new importations of EVD into the country until transmission in the entire West African sub-region was stopped. The risk of a new emergence from an animal reservoir, importation, sexual transmission or a missed transmission chain led Liberia to enter a period of enhanced surveillance after the WHO declaration [7]. Subsequently, there occurred short-lived re-emergence of EVD in two small clusters involving six cases in June [8, 9] and three cases in November, 2015 [10]. Prior to the 2014 EVD outbreak in West Africa, there was no laboratory capacity to diagnose Ebola in Liberia. During the outbreak, laboratory capacity to diagnose EVD was established in-country and disease/outbreak confirmation became largely reliant on laboratory testing of whole blood, dead-body swabs, and post-mortem heart blood from suspected cases. Quantitative reverse transcription polymerase chain reaction (qRT-PCR) [11], the gold-standard diagnostic assay for detecting and quantifying Ebola virus, was predominantly used [12-14]. In 2015, the Xpert^®^ Ebola assay for the GeneXpert platform (Cepheid, Inc., Sunnyvale, CA, USA) [15] an RT-PCR based assay, received emergency use authorization for Ebola diagnosis from the United States Food and Drug Administration [15]. Decreasing numbers of patients in the EVD holding and treatment centers prior to the 42-day period led to a rapid reduction in the number of laboratory requests [1]. This ultimately led to closure of eight EVD testing laboratories leaving three functional laboratories. Transition into enhanced surveillance led to a change from acute testing for clinical triage to surveillance testing [13], in which the threshold for the case definition was lowered to effectively demonstrate the absence of EVD in the local population [1]. When prevalence of a disease is low, there is a need to test more suspects (enhance surveillance) in order to rule out infection [1]. This resulted in an increase in the number of specimens thus increased demand on the efficiency and capacity of the remaining laboratories to test the exponentially increasing volume of specimens. We report the effect of interventions to boost laboratory capacity during the high demand period of enhanced EVD surveillance from May to December, 2015, and share lessons learned and recommendations for consideration in future laboratory capacity enhancement.

## Methods

We analyzed laboratory and epidemiologic data generated daily between May and December, 2015. The Republic of Liberia is divided into five regions; North Eastern, North Central, South Central, South Eastern-A, South Eastern-B [16]; and consists of 15 administrative counties [3]. Approximately, 50% of Liberia’s population lives in Montserrado County in which the country’s capital city, Monrovia, is located [17]. Using archived data from WHO Emerging and Dangerous Pathogens Network (EDPLN) for EVD [18] hosted at WHO headquarters in Geneva, Liberia epidemiologic surveillance hosted at the Ministry of Health of Liberia, and individual laboratory log-books available at the respective laboratories, we obtained laboratory characteristics and trend of laboratory capacity before and after interventions. Interventions included: 1) establishing and developing EVD testing at two laboratories; 2) implementing new EVD diagnostic techniques not previously used in Liberia; 3) recruiting and training personnel in EVD molecular diagnostic procedures [13, 15]; 4) creating “partial testing shifts” to enable longer testing hours. Laboratories were characterized by name, location, date of establishment, date of closure, type, operating party, technology used to diagnose EVD and number of staff competent in EVD diagnostics.

Laboratory capacity was defined with respect to daily and surge testing capacity, turnaround time, number of EVD suspected specimens tested, and specimen backlog as a measure of the difference between the number of specimen received and number of specimen tested by the laboratories. Daily testing capacity was defined as the number of EVD suspected specimens tested per day. Surge testing capacity was considered to be the maximum number of EVD suspected specimens that could be tested per day. Turnaround time was defined as the time taken by each laboratory to generate and disseminate test results to the Ministry of Health leadership, from the time a specimen was received. All the six laboratories that conducted EVD diagnosis, at some point, during the period of enhanced surveillance were included in the analysis. Other laboratories involved in clinical or public health diagnostics but not EVD diagnosis were not considered. Using archives from the epidemiologic surveillance database, we extracted data on number of specimens received, and number of specimens tested per laboratory per day. We also obtained the number of specimen that did not get tested the same day they were received in a given laboratory and defined this as specimen backlog. Non normally-distributed continuous variables were summarized by median and interquartile range while categorical data were summarized as proportions. We reported the trend of number specimen tested and specimen backlog per month. Stata 14 (Stata Corporation, College Station, TX, USA) was used for all analyses. This was a retrospective analysis of data as part of documentation of best practices, and did not necessitate ethical approval; however, the use of data, analysis and report were approved by Ministry of Health, Liberia, and the WHO Liberia Country Office.

## Results

**Figure 1:**
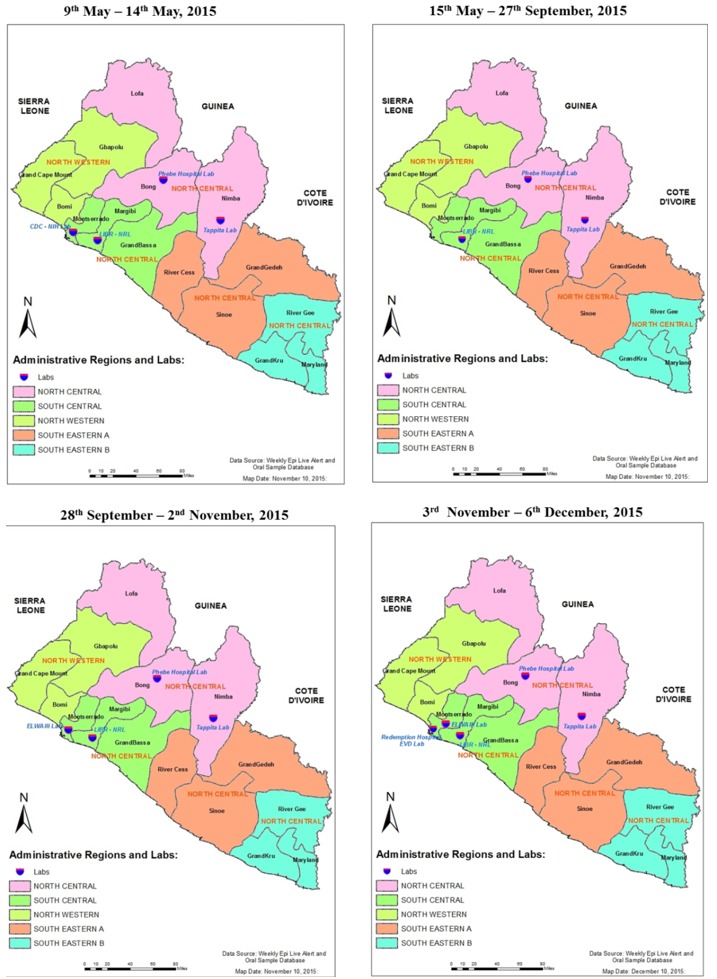
Distribution of Ebola Virus Disease testing laboratories per county and administrative region during heightened surveillance in Liberia, 2015

**Country laboratory coverage:** at the start of enhanced surveillance in May 2015, Liberia had a total of four EVD testing laboratories (Table 1) representing 40% (2 out of 5) and 26.6% (4 out of 15) regional and county coverage, respectively (Figure 1). From 15th May until the peak of the enhanced surveillance in September-October, 2015, the country had three EVD testing laboratories following closure of one. This led to reduction in the overall country coverage to 20% (3 out of 15). These laboratories employed qRT-PCR technology for diagnosis (Table 1).

**Table 1:** Characteristics of Ebola Virus Disease testing laboratories during enhanced surveillance in Liberia, 2015

EVD Laboratory	County	Date started Operating	Date closed	Facility type	Technology used	No. of staff	
						Median	Range
**LIBR – NRL**	Margibi	7-Aug-14	Open	Renovation	qRT-PCR GeneXpert	4	4,7
**CDC-NIH**	Montserrado	28-Sep-14	15-May-15	Renovation	qRT-PCR	0	0,0
**Phebe Hospital**	Bong	3-Oct-14	Open	Renovation	qRT-PCR GeneXpert	4	4,6
**JFD Hospital**	Nimba	5-Dec-14	Open	Renovation	qRT-PCR BioFire Film Array GeneXpert	4	2,6
**ELWA III**	Montserrado	28-Sep-15	Open	Mobile	GeneXpert	4	4,4
**Redemption Hospital**	Montserrado	3-Nov-15	Open	Renovation	GeneXpert	6	6,6

**LIBR-NRL:** Liberia Institute for Biomedical Research – National Reference Laboratory

**qRT-PCR:** Qualitative reverse transcriptase polymerase chain reaction

**GeneXpert:** Ebola GeneXpert Assay

**Establishment of laboratories:** to improve country-wide laboratory coverage, a mobile laboratory was successfully re-established at ELWA Ebola treatment unit (ETU), ELWA III laboratory, in Montserrado County and opened on 28th September, 2015 (Table 1). This restored the regional and county laboratory coverage to 40.0% and 26.6%, respectively (Figure 1). On 3rd November, 2015, Redemption Hospital clinical laboratory, located in Montserrado County, having completed the proficiency testing program, began testing whole blood for EVD, exclusively on samples from patients attending the hospital.

**Implementation of new diagnostic techniques:** due to a need to increase unit output, 10 GeneXpert machines with EVD testing capacity were implemented, with support from The foundation for innovative new diagnostics (FIND), WHO, academic consortium combating Ebola in Liberia (ACCEL) and United States Centers for Disease Control and Prevention (CDC), in Liberia. Four machines were installed at ELWA III laboratory in September 2015, one was installed at Redemption hospital laboratory in August 2015, two were installed at Phebe Hospital EVD laboratory in November 2015 and three was installed at Jackson F. Doe (JFD) Hospital EVD Laboratory in November 2015. The National Reference Laboratory (LIBR-NRL) partly used two GeneXpert machines that belonged to the National Institute of Health (NIH) research laboratory located at the Liberia Institute of Biomedical Research (LIBR) premises. Plans to establish a new EVD testing site, to use GeneXpert, in Maryland County, South Eastern-B region, were pending implementation by the time of this report. Implementation of the OraQuick^®^ Ebola Rapid Antigen Test (OraSure Technologies, Inc.) was in the process of being rolled-out at the time of enhanced surveillance and is therefore not discussed further.

**Recruiting and training of personnel:** between May and July 2015, there were 10 Liberian laboratory technicians competent in EVD diagnosis. Starting in August 2015, 16 technicians were recruited and trained in EVD molecular diagnostics. By October, 15 of them were competent in EVD diagnostic techniques. An additional 4 personnel were trained specifically on use of Xpert Ebola technology for Ebola diagnosis. By mid-October, there were up to 29 laboratory technicians proficient in EVD diagnosis (Table 1). International personnel with expertise in Ebola diagnosis were also recruited throughout the period to support diagnosis and training of local personnel. Earlier attempts to recruit more international experts by WHO and other partners, were not successful, due to the long procedures involved in international personnel recruitment and the lack of a database of experts to recruit from.

**Implementing partial testing shifts:** ELWA III laboratory established time-based shifts in which technicians reported and left work at different times, with considerable intersection between shifts. Phebe and JFD Hospital EVD laboratories had staff serving both the clinical and EVD laboratories. The work roster between the two laboratories was adjusted to cover the extended testing hours required for dedicated EVD testing. The Laboratory Manager would typically receive calls from a courier Riders for Health alerting him on the expected time of delivery of specimens. This notification would then trigger an action plan with assignment of staff to each stage of testing pipeline (sample inactivation, DNA extraction to RT-PCR or Biofire Film Array) to availing the results to the data manager. The staff assignment to a particular step or steps was based on their level of proficiency and comfort in carrying out the task in a timely manner and with accuracy regardless of the number of specimens. Bong EVD lab and NRl/LIBR established and published the cut-off time for sample reception and same day testing. The JFD EVD lab in Tappita normally received specimens towards the end of the official working hours so the laboratory maintained long opening hours. The Xpert Ebola Assay was introduced in these two laboratories when real-time EVD surveillance had been regained, that is to say, after all backlogged specimens had been tested and results disseminated). In addition, in July 2015, the JFD EVD laboratory began conducting semen testing for EVD, two days per week, in support of the National Men’s Health Screening Program thus requiring further adjustment in the working schedule.

**Figure 2:**
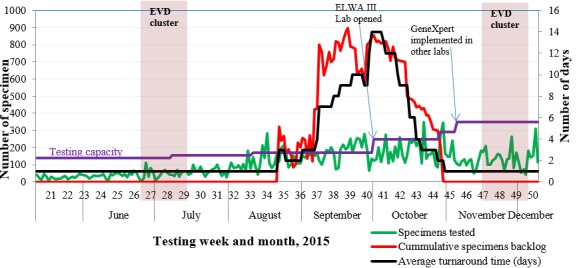
Laboratory capacity indicating specific intervention points during enhanced surveillance in Liberia, 2015

**Overall impact:** the median daily and surge testing capacity averagely increased from 35 and 102 to 77 and 134 specimens, respectively, excluding Redemption hospital EVD testing laboratory (Table 2). There was a 4-fold increase from daily to surge testing capacity (Table 2). The testing turnaround time had increased to 14 days by September 2015; however, it was reduced to within a day by the end of October 2015 (Figure 2). The total number of specimens tested for EVD increased from 651 specimens in May to 5,790 (88.9%) specimens in October (Table 3). An increasing specimen backlog (specimen that did not get tested for EVD within a 24 hours from the time of receipt at the respective laboratories) was observed from August 2015, with a peak of 896 specimens by mid-September 2015. The increase in the specimen backlog correlated with the increase in the total number of specimens received by the laboratories. This backlog decreased by end of October to 0 (zero) specimens, approximately three weeks prior to the November 2015 EVD flare in Liberia (Figure 2).

**Table 2:** Daily and surge capacity, and turn-around time of Ebola Virus Disease testing laboratories during enhanced surveillance in Liberia, 2015

Laboratory	Daily Testing Capacity		Surge Testing Capacity/day		Turn-around Time in days	
	Median	Range	Median	Range	Median	Range
**LIBR-NRL**	84	60,120	140	132,175	3	0,14
**Phebe Hospital**	20	8,32	56	40,62	2	0,14
**JFD Hospital**	58	14,58	120	116,174	0	0,5
**ELWA III**	80	60,100	120	120,126	0	0,1
**Redemption Hospital**	1	0,9	16	16,16	0	0,1

**LIBR-NRL:** Liberia Institute of Biomedical Research – National Reference Laboratory

**CDC-NIH EVD** Laboratory was not included in this table because it was closed six days into enhanced surveillance

**Table 3:** Number of Ebola Virus Disease suspected specimen tested and specimen backlog between May and December, 2015, in Liberia

Month	No. of specimen tested per day		Total No. of Specimen tested	Specimen backlog	
	Median	Range		Median	Range
May*	30	9,46	651	0	0,0
**June**	40	3,111	1208	0	0,0
**July**	60	28,111	1897	0	0,0
**August**	114	42,282	3982	0	0,266
**September**	183	65,281	5206	772	120,896
**October**	166	84,350	5790	442	0,819
**November**	125	49,264	3726	0	0,0
December*	148	42,312	947	0	0,0

* In this analysis, May starts from 9^th^ to 31^st^ and December starts from 1^st^ to 6^th^. All other months are complete

## Discussion

In our review of interventions used to boost laboratory capacity in Liberia following the 2014 and 2015 EVD outbreaks, we found that a combination of increasing the number of laboratories with EVD testing capacity by two, introduction of a new faster diagnostic technique, increasing the number of competent staff and increasing hours of testing, tremendously increased the overall laboratory output during enhanced surveillance and enhanced the country’s response capacity and effectiveness. Closure of one of the EVD laboratories in mid-May, 2015, was a result of greatly diminished demand for laboratory services due to decreased numbers of patients in EVD holding and treatment units between February and March, 2015 [1]. Montserrado County was the ideal location for establishing new EVD testing sites because it is the most populous county, yielded the highest number of specimen, had one functional Ebola treatment unit (ETU) located at ELWA III hospital, and yet did not have an EVD testing laboratory [19]. Although testing capacity was limited in the South-Eastern area of Liberia (Maryland County) [16], it was not possible to establish another EVD testing site within a short time mainly due to lack of essential components of a field EVD testing laboratory including glove-box and limited human resources. However, this is achievable given more time to establish testing capabilities and trained workforce. GeneXpert was chosen as the diagnostic system to be implemented as it is a closed system with lower biohazard risk, offered an opportunity to increase laboratory output in a short time due to its shorter turn-around-time, it is less labor intensive compared to conventional qRT-PCR, requires minimal technical knowledge to operate and thus required less time to train technicians, and operates up-to 45 cycles allowing for more sensitive detection compared to the conventional RT-qPCR [15, 20].

Under the existing circumstances, it took some time for the impact of recruiting and training new local staff in EVD diagnostics to be felt as developing competence in qRT-PCR techniques takes approximately one and a half months or more if the trainees have no theoretical knowledge of molecular diagnostics. Upon gaining this competence, at approximately two and a half months from the time of recruitment of the additional staff, a boost in the overall output was registered. The international staff also provided a significant boost in EVD testing capacity. Establishing testing shifts at laboratories allowed for more productive testing hours. Establishment of testing shifts coupled with implementation more rapid Xpert Ebola assay allowed for increased testing output. It takes approximately 2 hours to obtain results using GeneXpert due to complete integration and full automation of the process, compared to 4 to 6 hours using conventional qRT-PCR that is affected by long pre-analysis stage (sample inactivation and extraction). LIBR-NRL and Phebe Hospital EVD laboratories had longer turn-around-time (Table 3) because they received more samples than other laboratories due to availability of more storage space for unprocessed specimens, and accessibility throughout the year. During the rainy season, accessibility of JFD hospital EVD laboratory was greatly compromised and therefore specimens were redirected to Phebe hospital EVD lab. This increased the volume of specimens received by the laboratory hence the increased turn-around-time observed. ELWA III lacked storage capacity for unprocessed samples and therefore always forwarded excess samples to LIBR-NRL. Following the outbreak of the second EVD cluster that originated in Margibi County [16] at the end of June, 2015 [8], the number of specimens received by laboratories greatly increased (Figure 2). This was attributed to more pro-active surveillance activities by all stakeholders. By mid-August 2015, the number of specimens had increased beyond the overall laboratory testing capacity thus yielding a cumulative specimen backlog. Having increased the number of laboratories, competent staff, working hours and implemented GeneXpert testing; the daily and surge EVD testing capacity increased thus increasing the number of specimen tested, decreasing the specimen backlog and yielded a reduction in testing turn-around time. We also observed that the number of specimens from EVD suspects collected from around the country, generally decreased as requests were sent to the health facilities to adhere to the screening criteria for EVD using the surveillance EVD case definition [1], and is believed to have partly contributed to the reduction in the specimen backlog. The new cluster of EVD cases observed in November, 2015 came approximately three weeks after clearing specimen backlog and at a time when laboratory capacity was sustainably efficient at approximately 350 specimens a day and turnaround time had been restored to less than 24 hours. This greatly facilitated the quick release of results which was essential to mobilize appropriate resources to contain the outbreak in a timely manner. As such, this cluster involved only three confirmed cases and it lasted approximately two weeks from admission of the first case to the ETU to discharge of the last case from the ETU. In addition, enhancing the laboratory capacity enabled reinstating of public health diagnostic services beyond testing for EVD especially for some of Liberia’s Integrated Disease Surveillance and Response (IDSR) priority diseases. Re-instated capacity included measles and rubella in-country testing, Lassa fever and acute flaccid paralysis referral to international/regional reference laboratories. Recruitment and training of new technicians in EVD testing allowed for resumption of measles and rubella testing by the competent technicians who had previously been taken up with EVD diagnosis. Implementation of the EVD assay on the GeneXpert platform enabled integrated testing for tuberculosis, HIV viral load and HIV early infant diagnosis using the same instruments at JFD, Redemption and Phebe Hospitals.

### Limitations

This is a documentation of best practices and not a research study, therefore, data used was obtained retrospectively. As a result, some data presented are estimates from a range recorded in the data sources. We have, however, included these ranges in the results to ensure accuracy of the data presented.

## Conclusion

A combination of opportunity and supporting measures was adjusted to be responsive to the prevailing circumstances in Liberia with the aim of enhancing and maintaining of laboratory capacity for timely EVD diagnosis. These included leveraging available resources; maximizing the testing capacity at each laboratory using existing and new diagnostic platforms and supplies; emergency procurement of supplies and reagents to meet the demand; effective coordination and monitoring of testing at each laboratory; better forecasting and re-budgeting to stabilize the demand-testing equilibrium; and deploying additional staff. Enhancing and retaining local capacity and competencies to respond to any disease outbreak cannot be underscored. Given the possibility of reemergence of disease clusters or future outbreaks; a well-trained, competent and motivated workforce will enable continuity of laboratory services for disease surveillance, routine patient services and sustained vigilance for emerging and re-emerging disease threats. Data-driven decision trees should be used by all stakeholders to inform suspension or scaling down of essential laboratory services during a disease outbreak. This strategy is necessary to ensure that such services can be easily reinstated or re-scaled upwards during or after future disease outbreaks. Establishing key services during any given disease outbreak or crisis by governments, partners and other stakeholders should preferably be done by incorporating these services into already existing structures and involving local staff to enable sustainability and longevity. A robust supply chain and inventory system as well as budgeting and forecasting mechanisms by responsible parties to prevent shortage of essential reagents and other consumables are central to providing consistent and reliable diagnostic services.

### What is known about this topic

- There is limited laboratory capacity especially in developing countries affecting timely diagnosis and therefore response to epidemic-prone diseases;
- Development of laboratory capacity in such settings usually takes a lot of time and requires a lot of resources.

### What this study adds

- This paper demonstrates how a combination of context specific interventions can rapidly enhance laboratory capacity in a cost-effective manner, especially during times of abrupt high demand.

## Competing interests

The authors declare no competing interest.
